# Future and Present Hedonistic Time Perspectives and the Propensity to Take Investment Risks: The Interplay Between Induced and Chronic Time Perspectives

**DOI:** 10.3389/fpsyg.2018.00920

**Published:** 2018-06-05

**Authors:** Katarzyna Sekścińska, Joanna Rudzinska-Wojciechowska, Dominika Agnieszka Maison

**Affiliations:** ^1^Faculty of Psychology, University of Warsaw, Warsaw, Poland; ^2^Wroclaw Faculty of Psychology, SWPS University of Social Sciences and Humanities, Wroclaw, Poland

**Keywords:** chronic Time Perspective, induced Time Perspective, risk, investment portfolio, financial risk-taking

## Abstract

Willingness to take risk is one of the most important aspects of personal financial decisions, especially those regarding investments. Recent studies show that one’s perception of time, specifically the individual level of Present Hedonistic and Future Time Perspectives (TPs), influence risky financial choices. This was demonstrated for both, Time Perspective treated as an individual trait and for experimentally induced Time Perspectives. However, on occasion, people might find themselves under the joint influence of both, chronic and situational Time Perspectives and little is known about interactions between them. The paper focuses on the interplay between chronic and induced levels of Future and Present Hedonistic TPs in explaining people’s propensity to take investment risks. An experimental study using a Polish national random-quota sample was conducted. The results showed that situationally induced Future TP lowered the preferred level of portfolio riskiness while situationally induced Present Hedonistic TPs resulted in exactly the opposite effect, and that the higher level of chronic Present Hedonistic TP was linked to higher investment risk preferences. The role of the chronic Present Hedonistic TP was moderated by the situationally induced Future (approaching significance) and Present Hedonistic TPs. The induction of these TPs resulted in reduction of the propensity to take investment risks. The study adds to the literature on psychological factors influencing the propensity to take financial risk. The results are also important for researchers who experimentally manipulate variables that might be also considered as chronic traits. They indicate that whether the manipulation is congruent with one’s natural tendencies may have a differential impact on subsequent measures.

## Introduction

Willingness to take risk is one of the most important aspects of personal financial decisions, especially those involving investments. The propensity to make risky decisions have been a subject of interest to many researchers, also from the field of psychology. As a result of their investigations into the role of psychological factors that influence people’s risky choices, it has been shown that the willingness to take financial risk is affected by numerous individual variables, such as narcissism (Foster et al., 2009), sensation seeking, and locus of control (Wong and Carducci, 2016) or motivational system (Sekścińska et al., 2016). However, apart from individual traits, people’s behavior, or way of thinking, can be influenced by more or less subtle environmental cues, such as advertisements, conversations with others and many more. Research shows that he propensity to make risky financial decisions may also be influenced by the situational external factors, for example framing (e.g., Tversky and Kahneman, 1981), fresh memory of success (Forgas, 1995), and other positive memories, e.g., those related to previous wins (Ludvig et al., 2015), situationally induced promotion, or prevention focus (Sekścińska et al., 2016), familiarity with specific tasks (Massa and Simonov, 2006), mood (Leith and Baumeister, 1996), or even the activation of a different social role (Sekścińska et al., 2016).

In the present study, we focus on people’s Time Perspective (TP) as a determinant of the propensity to take investment risks. We take into account chronic level of TPs as well as situationally induced TPs and analyze an interplay between them.

Investing money entails a choice between consuming less today in order to possibly increase one’s wealth in the future and spending now and facing a lower level of wealth in the future. This kind of decision is an example of an intertemporal choice. It involves tradeoffs between consequences (positive and negative) that will occur at different points in time (Frederick et al., 2002). Generally, present rewards are preferred over later ones, a phenomenon called delay discounting, and the amount of future incentive necessary to match the value of the immediate reward is an individual difference (Kirby and Maraković, 1996). Some individuals are characterized by “steep” discounting rates, which means that the value of future rewards depreciate quickly and a large additional future reward is necessary to offset the value of the present one. Others discount future rewards slowly (“shallow” discounting rate) and need only a small additional future reward in order to match the value of the present one. Studies show that the propensity to wait for delayed outcomes is influenced by such individual variables as personality factors (Funder et al., 1983) or cognitive abilities (Shamosh and Gray, 2008). It can also by situationally modified, for example by framing (e.g., the hidden zero effect, Magen et al., 2008), change of mental construal (Fujita and Han, 2009; Rudzinska-Wojciechowska, 2017), or even encountering a “mating opportunity” (Wilson and Daly, 2004).

The ability to delay gratification is often linked with TP because the tendency to wait for the delayed outcomes is the core feature of this construct, especially Future TP and Present Hedonistic TP – characterized by deficits in this area (Zimbardo and Boyd, 2008). Both constructs relate to how an individual perceives tradeoffs between present and future (Daugherty and Brase, 2010). Consequently, TP should theoretically impact decisions involving choices between present and future rewards. Indeed, studies investigating this relationship show that the two variables are similar but non-redundant (Daugherty and Brase, 2010).

Another important aspect of investment decision is the amount of risk one is prone to accept. Numerous studies have confirmed that the propensity to take risks can be linked to one’s Time Perspective (Zimbardo et al., 1997; Keough et al., 1999; Henson et al., 2006). Risky financial choices are no exception. They were shown to be influenced by one’s perception of time. Studies indicated that two TPs have a crucial role in explaining this kind of decisions, namely Present Hedonistic and Future TPs (Jochemczyk et al., 2017; Sekścińska et al., 2018). Interestingly, both, chronic level of TPS and experimentally induced TPs were shown to impact risky financial choices (Sekścińska et al., 2018). However, it is unclear how the induced and chronic TPs interact and influence risky financial decisions. For example, what happens when someone scoring low regarding the Present Hedonistic TP encounters a message which activates values represented by this TP (e.g., joy of living the moment)? And what are the consequences of activating in this person a different TP (for example, a Future one)?

There is a mall albeit steadily growing body of research indicating that individual’s chronic traits can interact with situational activation of corresponding states. They suggest that chronic traits moderate the effect of situational manipulations on subsequent decision making (for example Jain et al., 2007; Haws et al., 2012). This issue hasn’t been investigated in the context of Time Perspective yet, as the attempts to induce particular Time Perspectives in experimental conditions are new in the field. At the same time, understanding this moderation effect is significant. If effects are only operating among certain groups of participants or even acting in an opposite way among a group of them, then null results may be found as a whole, even though significant differences do exist (Wheeler and Berger, 2007). In this paper, we will focus on the interplay between chronic and induced TPs in explaining people’s propensity to take investment risks.

### Time Perspectives

A TP is a psychological construct that represents an individual’s relationship with time (Zimbardo and Boyd, 1999). One of the most important theories in the area is Zimbardo and Boyd’s (1999, 2008) Time Perspective Theory. It posits that one’s perception of time influences decision making by locating the primary set of psychological influences within the temporal frames of either the present, the past, or the future (Zimbardo et al., 1997). Zimbardo and Boyd (1999) distinguish five TPs: Past Negative, reflecting an aversive view of the past; Past Positive, characterized by a warm, sentimental attitude toward the past; Present Hedonistic, which is characterized by an orientation toward present pleasure, risk, and enjoyment; Present Fatalistic, related to a helpless and hopeless attitude toward one’s future, and Future, related to behavior dominated by striving for future goals and rewards. One’s perception of time was found to be a relatively stable individual difference trait, although it is believed that it can be intentionally and situationally modified (Zimbardo and Boyd, 1999, 2008). A recent study conducted in our laboratory showed that induced TPs lead to a similar pattern of results to those obtained in studies on chronic TPs (Sekścińska et al., 2018).

### Time Perspectives, Financial Choices, and Personal Investment Decisions

Although individual differences in TPs have been associated with affective, cognitive, and behavioral outcomes (e.g., subjective well-being, Zhang et al., 2013), their role in financial choices is not well established. Zimbardo and Boyd (2008) predicted numerous associations between TPs and financial behaviors, but there has not been much research in this area. Nevertheless, the existing studies indicate that this link is worth investigating. For instance, it has been shown that people’s financial health correlates strongly with their approach to time (Clements, 2014). Furthermore, previous studies conducted in our laboratory indicate that people’s saving and investing preferences are related to their TPs (Sekścińska, 2014; Sekścińska et al., 2018).

Numerous studies have confirmed the link between the propensity to take risk and TPs, showing that more future-oriented and less present-oriented individuals display fewer risky behaviors. Individual differences in time perspective have been associated with risky behaviors such as risky driving (Zimbardo et al., 1997) tobacco, alcohol and illegal drug use (Keough et al., 1999) and risky health behaviors (Henson et al., 2006). Nonetheless, there is only a handful of studies focusing on the role of TPs in risky financial decisions and investment choices. Meanwhile, while analyzing the propensity to take risk, one must bear in mind that it is domain specific (Slovic, 1972; Weber et al., 2002) and people might seek risk in one domain (for example health) and avoid it in other (for example social or financial).

In the recent study by Jochemczyk et al. (2017), the Present Hedonistic TP correlated with the propensity to take risks in various domains, including financial. At the same time, the Future TP was negatively correlated with risk taking in various domains, except in the financial/investing and social domains. The authors argue that the lack of relationship between chronic Future TPs and investment risk taking can be explained by Zaleśkiewicz’s (2001) theory of instrumental and stimulating risk taking. This theory posits that there are two distinct forms of economic preference which differ with the basic motives that stimulate risk taking. People take instrumental risk in order to reach a future profit. While doing so, they avoid situations which cannot be controlled. They are also not interested in a feeling of arousal associated with risky behaviors. People who prefer taking stimulating risk, on the other hand, perceive this arousal as pleasant and seek this state. They also look for immediate sensations and excitement. The author of the theory demonstrated that instrumental risk is related to risk preference in the investment domain while stimulating risk is related to the preference for recreational, ethical, health and gambling risks. According to Jochemczyk et al. (2017), the risk related to investing in stocks or bonds is instrumental, while, according to the definition of Future TP, this Time Perspective is more likely to be negatively related to stimulating risk preferences, not instrumental ones. However, it has to be noted that the investment risk in this research was understood as both investments in stocks and new businesses.

The pure investment risk was investigated in the latest study of Sekścińska et al. (2018), which confirmed the role of TPs, especially Present Hedonistic and Future, in explaining people’s financial choices, particularly their propensity to consume, save, invest, and take investment risks. The results of the studies showed that the Higher Future TP was related to a propensity to invest and make safe investment choices, while the Higher Present Hedonistic TP was related to a low propensity to invest and a preference for risky investments. Additionally, the abovementioned research project was, to our best knowledge, the first published attempt to induce TPs in experimental conditions and it demonstrated that situational TPs lead to similar patterns of results to those obtained in studies on chronic TPs. However, the influence of situational and chronic TPs on financial decisions was investigated in two separate studies. Therefore it was not possible to examine the interaction between them.

### Chronic Traits and Situational Activation – The Interplay

Studies usually focus either on chronic levels of given traits or situationally induced states. The interaction between these two is seldom investigated. Meanwhile, each time a state is situationally manipulated, every participant has extant underlying levels of a given chronic trait (Haws et al., 2012). As a result, experimental manipulations might have different effects for different people based upon underlying associations or chronic tendencies possessed by the individual (Wheeler and Berger, 2007). For example, Wheeler and Berger (2007) demonstrated that the same prime – thinking of attending a party – made introverts to be more likely to select “comfort” products when compared to extroverts. Similarly, Pillaud et al. (2015) tested whether stereotypical situations would affect low-status group members’ performance more strongly than high-status group members. They used gender as a proxy of chronic social status and a gender-neutral task that was randomly presented to favor boys or girls. The results showed that girl’s performance suffered more from the evoked stereotypes than did boy’s one. Thus, exploring the interplay between one’s chronic traits and situationally induced states might broaden our understanding of subsequent decisions. Moreover, being unaware of this kind of effect might lead to a failure in finding significant relationships even when they exist (Haws et al., 2012).

There is only a handful of studies investigating the interplay between traits and corresponding situationally induced states. Due to the fact that they are conducted in various domains and not all of them focus directly on the issue of the interplay between chronic and situational influences on a task or decision, it is hard to generalize their results. Nevertheless, they show that chronic traits and situational manipulations interact.

Some studies show that that chronicity amplifies temporary effects because of greater susceptibility to external primes. In a study examining the interactions between chronic and situational promotion and prevention focus, Haws et al. (2012) demonstrated an asymmetric effect in the interactions. Specifically, the effects were amplified when chronic promotion focus was paired with a promotion manipulation, but not when chronic prevention focus was paired with a prevention manipulation. In a study focusing on the same domain, similarly, Keller and Bless (2006) investigated the impact of chronic and situational self-regulatory mechanisms on cognitive test performance and demonstrated that the test performance was enhanced when situationally induced regulatory mechanisms matched the chronic self-regulatory focus of a participant as compared to the mismatch conditions.

On the other hand, there are some studies which demonstrate that the priming effect is redundant with the same chronic tendency. In a research investigating the effect of chronic and temporary sources of accessibility on impression formation, Johar et al. (2003) hypothesized that gender stereotypical primes will have little influence on subsequent judgments of those with a medium or high tendency to gender stereotype (due to their redundancy) but gender stereotypical primes will result in the classic assimilation effect for those with a low tendency to gender stereotype. They tested it in the domain of female role portrayals in advertising and examined the effect of advertisements that featured a stereotypical prime – women as homemakers (vs. did not feature women) on trait judgments of a target woman whose behaviors were ambiguously described. The results of the study confirmed the redundancy hypothesis: judgments of medium and high tendency to stereotype participants were not affected by advertisements portraying homemakers. Also as expected, judgments of low tendency to stereotype participants were assimilated to the homemaker prime. A similar pattern of results was obtained in a study conducted in a context of cultural analysis by Gardner et al. (1999). The researchers primed American (vs. Hong Kong) participants with interdependent (independent) self. The shifts in values were observed only in those conditions in which the self prime was different from what was chronically encouraged by the cultural context. Members of a culture that chronically encouraged an independent self-construal remained unaffected by the independence prime, but shifted their value endorsements to reflect relatively more relationship- and group enhancing goals in response to an interdependent prime. In contrast, participants in a culture that chronically encourages interdependent self-construals remained unaffected by the interdependence prime, but shifted their value endorsements to reflect relatively more individualistic goals when primed with independence.

The evidence presented above highlights the importance of research on interactions between chronic traits and corresponding induced states. Unfortunately, the nature of those mechanisms is not entirely clear, as some studies show that the effects of situational influence are amplified by chronic traits whereas other indicate that chronicity masks temporary effects because of redundancy. Nevertheless, they all demonstrate that when a variable is investigated both as a chronic trait and experimentally induced state, it is important to examine the interplay between them.

## Current Study

Previous studies indicated that Present Hedonistic and Future TPs play a particularly important role in explaining people’s risky investment decisions. This was shown for both chronic and situational level of these TPs. However, the studies did not take into account the interplay between TPs understood as a personality trait and those situationally induced. We aim to investigate the nature of this relationship.

An experimental study was conducted in order to examine the role of chronic levels of Future and Present Hedonistic TPs in people’s propensity to take investment risks depending on the situationally induced Future and Present Hedonistic TPs. Moreover, the study verifies the direct role of chronic and induced Present Hedonistic and Future TPs in the propensity to take investment risk.

Using the theoretical framework of Zimbardo and Boyd (1999, 2008) as well as basing on the results of previous studies, which demonstrate the link between TPs and financial decisions (Jochemczyk et al., 2017; Sekścińska et al., 2018) and show moderating role of chronic traits on situational factors (Jain et al., 2007; Haws et al., 2012) we elaborated the following hypotheses:

(H1) Present-hedonistic-oriented people prefer risky investment portfolios.
(H2) People with high levels of chronic Future TP prefer safe investment options.
(H3) Induced TPs influence people’s investment risk preferences. Induced Future TPs lower people’s preferred level of investment risk while induced Present Hedonistic TPs heighten people’s financial risk preferences.
(H4) There is also a moderation role of induced TPs in the relationship between chronic TPs and financial risk preferences. However, given the lack of studies on induced TPs and mixed results of studies focusing on the interaction of chronic traits and corresponding situational states, we expect one of two possible effects. The activation of TPs result either in

(1) decisions in accordance with the induced TP; namely, the tendency to choose safer investment portfolios after induction of Future TP and the preference for more risky investment portfolios after induction of Present Hedonistic TP
or
(2) decisions consistent with chronic TP but more extreme; encountering messages incongruent with one’s temporal preferences might result in the tendency to defend personal beliefs.

The first prediction is based on previous research on the interactions of chronic and situational variables, which demonstrated that the effect of situational influences is usually noticeable, in both, congruent and mixed conditions. However, previous results are mixed and somewhat contradict each other. Therefore we base our second prediction on the well documented finding in the area of social psychology indicating that some persuasive messages might lead to unintended consequences and result in an enhancement of recipient’s original attitudes (Hovland et al., 1953, 1957; Martin et al., 1990). This phenomenon is called a boomerang effect and is more likely to occur when the communicator’s position is very far from the recipient’s position (Hovland et al., 1953). The change of attitudes in the opposite direction than intended (the boomerang effect) is often explained by a reactance theory (Brehm, 1966). It posits that when a person thinks that his freedom to support a position is being limited, the psychological reactance will be aroused and the position, view or attitude will be strengthened contrary to what was intended by a communicator. Thus, basing on the abovementioned findings, we assume that a person exposed to a given TP activation (for example Present Hedonistic) might feel that his or her other TP orientation (for example Future) is being restricted and, as a consequence, strengthen the original attitude. What is more, the feeling of restricted freedom might be the strongest among the participants who score higher on a given scale.

## Materials and Methods

### Participants

The study was conducted on internet research panel on nation-wide random-quota sample. The participants were randomly selected from the panel users and demographic structure of the sample was controlled in order to make it compatible with the structure of Polish population. The quotas were selected based on the distribution of gender, age, education, and size of the living place in the population of Poles. A total of 1,227 people participated in the study (701 women; 526 men), aged 18–78 years (*M* = 42, *SD* = 14.35). All participants provided their informed consent to take part in the research after reading a detailed information about the study. Participants were asked to click on a link to the study if they agreed to take part in the research. Otherwise, they did not participate in the study. All participants of the panel are rewarded for their participation in every study with points, which can be exchanged on rewards of their choice. The amount of points that is awarded for a particular study depends on its length. The Ethics Board of University of Warsaw Faculty of Psychology approved the study.

### Measures

#### Chronic Present Hedonistic and Future Time Perspectives

Chronic Present Hedonistic and Future TPs were measured using the short version (15 items) of the Zimbardo Time Perspective Inventory (SZTPI), created by Zhang et al. (2013). The items in SZTPI refer to the five TPs, and each TP is represented by three items. For example, an item measuring the Future TP is “When I want to achieve something, I set goals and consider specific means for reaching those goals.” Participants are asked to answer the question: “How characteristic or true is this of me?” on a 5-point scale: from 1-very untrue to 5-very true. The score of each TP is counted as the sum of the answers to the questions from the appropriate scale and ranges from 3 to 15.

#### Present Hedonistic and Future TPs Activation

The Present Hedonistic and Future situational TPs were activated using two versions of a manipulation task^1^. Each of these consisted of two sets of three sentences that the participants were asked to read (see: Sekścińska et al., 2018). The sentences were created based on the Zimbardo Time Perspective Inventory items (Zimbardo and Boyd, 1999). Each version of the manipulation task referred to the definition of a particular TP (e.g., Future TP: People believe that meeting deadlines and performing the necessary actions takes primacy over current entertainment, etc.)^2^.

After reading each set of sentences, the participants were asked to rewrite one of the sentences to enhance the effect of the manipulation. As a manipulation check, participants were asked to write three things that they remembered from the sentences they had just read. The results of the manipulation check showed that 97% of the participants from Present Hedonistic TP group and 98% of the participants from Future TP group wrote words consistent with the TP that was activated in their group. The other participants (in total 5 people) answered using neutral words (e.g., people, psychology, sociology, tests).

#### Propensity to Take Investment Risks

In order to measure the propensity to take investment risks, the participants were asked to create an investment portfolio by indicating what percentage of a hypothetical amount of money (PLN 10,000) they would want to allocate to a variety of financial market instruments. The participants could invest their hypothetical money in bonds, balanced mutual funds (investing 50% in stock and 50% in bonds), and stocks. The participants had the opportunity to select one or more of the instruments mentioned above. The task measured the propensity to take investment risks in terms of the general riskiness of the created portfolio (*riskiness of portfolio*) reflected by the percentage of shares (instruments that are affected by a significant risk of loss) in the portfolio. The indicator was based on the following formula: 0 × percentage of bond + 0.5 × percentage of fund + 1 × percentage of shares (with 0 indicating the safest portfolio, and 100 the riskiest portfolio). This authorial tool was previously used in several studies conducted by our team (Sekścińska, 2014, 2015; Sekścińska et al., 2016, 2018).

### Procedure

The study consisted of two stages. In the first stage, the participants were asked to complete the SZTPI questionnaire and provide sociodemographic data. After 1 week, an invitation to the next stage of the study was sent via the on-line panel to all 1227 participants of the first stage. They were not informed that the new invitation was related to the previous study. In the second stage of the study, 723 people took part. They were randomly assigned to one of three groups: the situational Present Hedonistic TP induction (*n*_1_ = 135), the situational Future TP induction (*n*_2_ = 106) or the third group in which none of the TPs were induced (*n*_3_ = 481^3^). After a particular TP had been activated (in both experimental groups) or at the beginning of the study (control group), participants completed the measure of propensity to take investment risk by allocating their resources to their portfolio. Finally, they were fully debriefed.

### Design of the Study

The levels (low or high) of chronic Present Hedonistic and Future TPs were the first and the second between-subjects independent variables. The induced TP was the third between-subjects independent variable (manipulation: Present Hedonistic TP, Future TP, none). The propensity for risky investment choices was the dependent variable.

## Results

The chronic Time Perspective variables were mean-centered prior to the regression analysis. The induced Time Perspective variable was recoded into two dummy variables. The first variable was Induced Present Hedonistic TP vs. others, coded as (1) Induced Present Hedonistic TP group, (0) other participants. The second variable was Induced Future TP vs. others, coded as (1) Induced Future TP group, (0) other participants.

Zero-order correlations between variables and scale properties are presented in **Table 1**. The significant relationships between riskiness of the created portfolio and Chronic Present Hedonistic TP (moderate positive), Induced Present Hedonistic TP vs. others (weak positive) and Induced Future TP vs. others (moderate negative). Moreover, the significant moderate negative relationship between two variables related to TPs induction was also found.

**Table 1:** Zero-order correlations between variables and scale properties.

Measures	*M*	*SD*	1	2	3	4	
1. Riskiness of portfolio *Scale from 0 to 100*	37.59	30.25					
2. Chronic Present Hedonistic TP *Scale from 3 to 15*	10.20	2.09	0.305**				
3. Chronic Future TP *Scale from 3 to 15*	12.18	2.13	-0.005	0.037			
4. Induced Present Hedonistic TP vs. others			0.267**	0.041	-0.055		
5. Induced Future TP vs. others			-0.331**	-0.049	0.008	-0.498**	

*^∗∗^p < 0.001.*

The stepwise multiple regression analysis was then conducted to investigate the role of chronic and situationally induced TPs as well as their interaction effects on predicting people’s propensity to build risky investment portfolios (**Table 2**).

**Table 2:** Predictors of riskiness of created portfolio.

	Step 1			Step 2			Step 3			Step 4		
	
Variables	*B*	*SE*	*p*	*B*	*SE*	*p*	*B*	*SE*	*p*	*B*	*SE*	*p*
Chronic Present Hedonistic TP (Ch_Pr_H)	4.33	0.73	<0.001	4.09	0.69	<0.001	0.93	1.23	0.452	0.615	1.27	0.627
Chronic Future TP (Ch_F)	-0.39	0.72	0.588	-0.11	0.67	0.869	-1.47	1.14	0.196	-1.262	1.16	0.277
Induced Present Hedonistic TP (I_Pr_H)				7.76	3.42	0.024	7.59	3.39	0.026	7.92	3.40	0.020
Induced Future TP (I_F)				-17.11	3.62	<0.001	-17.18	3.59	<0.001	-17.26	3.60	<0.001
Ch_Pr_H × Ch_F Ch_Pr_H × I_Pr_H Ch_Pr_H × I_F Ch_F × I_Pr_H Ch_F × I_F Ch_Pr_H × Ch_F × I_Pr_H Ch_Pr_H × Ch_F × I_F							-0.045 5.13 3.68 1.50 2.61	0.35 1.59 1.83 1.54 1.70	0.897 0.001 0.045 0.330 0.147	0.49 5.67 3.64 1.32 2.53 -1.01 -0.11	0.63 1.65 1.97 1.55 1.83 0.80 1.02	0.437 0.001 0.066 0.394 0.167 0.205 0.918
*F*	17.57		<0.001	22.62		<0.001	11.73		<0.001		9.77	<0.001
*R*^2^	0.09			0.20			0.23				0.24	

In Step 1 Chronic Present Hedonistic and Future TPs were introduced. The significant positive effect of Chronic Present Hedonistic TP on the riskiness of portfolio was found.

In Step 2 the variables coding TPs induction (Induced Present Hedonistic TP vs. others and Induced Future TP vs. others) were introduced. Both of them were found as an significant predictors of the portfolio riskiness although the effect was positive for Present Hedonistic TP induction and negative for Future TP induction. Moreover, after introducing the TPs induction variables the effect of Chronic Present Hedonistic TP was still found.

In Step 3 we introduced the two-way interactions between both chronic TPs and each of chronic TP and (a) Induced Present Hedonistic TP vs. others, (b) Induced Future TP vs. others. Both variables coding TPs induction were significant predictors of the portfolio riskiness (positive effect for Present Hedonistic TP induction and negative for Future TP induction). Moreover the significant interaction effects between Chronic Present Hedonistic and (a) Induced Present Hedonistic TP vs. others, (b) Induced Future TP vs. others were found. Simple slope analysis indicated that among people from the Induced Present Hedonistic TP group, the effect of Chronic Present Hedonistic TP was positive and significant, *B* = 6.06, *SE* = 1.04, *p* < 0.001, and positive but not significant among other participants, *B* = 0.93, *SE* = 1.23, *p* = 0.45.

Moreover, the simple slope analysis showed that among people from the Induced Future TP group, the effect of Chronic Present Hedonistic TP was positive and significant, *B* = 4.60, *SE* = 1.41, *p* = 0.001, and positive but not significant among other participants, *B* = 0.92, *SE* = 1.23, *p* = 0.45.

In Step 4 the three-way interactions between Chronic Present Hedonistic TP, Chronic Future TP and (a) Induced Present Hedonistic TP vs. others, (b) Induced Future TP vs. others were introduced. The significant effects of Induced Present Hedonistic TP vs. others (positive) and Induced Future TP vs. others (negative) were observed. Moreover, the significant interaction effect between Chronic Present Hedonistic and Induced Present Hedonistic TP vs. others was also found. Again, simple slope analysis indicated that among people from the Induced Present Hedonistic TP group, the effect of Chronic Present Hedonistic TP was positive and significant, *B* = 6.28, *SE* = 1.06, *p* < 0.001, and positive but not significant among other participants, *B* = 0.62, *SE* = 1.27, *p* = 0.63.

## Discussion

The goal of the research was to investigate the relationship between induced and chronic Future and Present Hedonistic TPs and the propensity to take investment risks. Previous studies demonstrated that chronic and situationally induced TPs are important factors influencing risky financial choices. Nevertheless, the interaction between these two was never demonstrated. In order to investigate its nature, an experimental study using a Polish national sample was conducted. It provided an evidence that the congruence of consumers’ chronic and situational TPs might have an impact on financial risky choices. Nevertheless, the relationship between TPs understood as an individual treat and treated as a situational variable is not straightforward.

As expected, the study showed the significant direct role of situationally induced Future and Present Hedonistic TPs. The induced Future TP lowered the preferred level of the portfolio riskiness, while the induced Present Hedonistic TP effect was exactly the opposite, which is in line with our expectations of the role of those TPs.

The results of the study did not confirm the role of chronic Future TP in explaining people’s propensity to build risky investment portfolios. This is in line with the results of the study by Jochemczyk et al. (2017) and may be explained by the instrumental and situational risk theory (Zaleśkiewicz, 2001) mentioned in the section “Introduction.”

The level of chronic Present Hedonistic TP had a direct effect on investment risk preferences. In accordance with our expectations, a high level of chronic Present Hedonistic TPs resulted in higher investment risk preferences. However, the role of the chronic Present Hedonistic TP seemed to be moderated by the situationally induced TPs. The Future TP induction lowered the riskiness of created portfolio of low chronic present hedonistic people. Those relationships were not observed when people were strongly Present Hedonistic. Moreover, the induced Present Hedonistic TP lowered the riskiness of portfolios of low Chronic Present Hedonistic people and increased the riskiness of portfolios among people with high level of that chronic disposition.

We expected that the induction of a particular TP would either result in a decision in accordance with this TP, or a decision consistent with a chronic TP, but more extreme. The first prediction turned out to be true in the case of the induced Future TP. Activation of this TP caused people to concentrate on the future consequences of current decision and highlighted the value of planning activities, which is difficult in the case of unpredictable share prices. Therefore, induced Future TPs lowered participants’ propensity to risky investment choices. The only exceptions were those who were strongly Present-Hedonistic-oriented. Probably, this group is not prone to think of the future, even if the Future TP is situationally induced, and do not change their risk preferences.

Nevertheless, activation of Present Hedonistic TP seems to lead to decisions consistent with chronic Present Hedonistic TP, but even more extreme. In the case of participants who had low levels of chronic Present Hedonistic TP, reading sentences so different to their own opinions probably led to changes in their preferences to more extreme and, as a consequence, they preferred even less risky investment portfolios. However, the moderation role of activated Present Hedonistic TPs seems to not be universal regarding chronic TPs, as there was no moderation role observed in the relationship between chronic Future TPs and financial risk preferences.

Our results contribute to understanding how TPs are linked with risky financial choices and specifically with the propensity to take investment risks. Therefore the study extends a growing literature on risk taking, financial decision making, and financial risk taking in particular. It also adds to previous findings on the role of situational factors that might influence consumer’s financial choices. It seems possible that even having a brief conversation, or seeing an advertisement just prior to the investment decision, might alter people’s choices and their preferences for risk. Moreover, this is, according to our best knowledge, the first study that aims to explore the relationship of chronic and induced TPs. There is no doubt that it extends the vast literature on TPs by confirming the influence of situationally induced Time Perspective on subsequent decisions and by shedding a light on the issue of the interplay between chronic and induced TPs. Moreover, although the present study focuses specifically on Time Perspectives, we are convinced that the implications of the research apply to a multitude of psychological constructs that are examined using both, experimental manipulations and psychometric methods. The results confirm that whether the manipulation is congruent with one’s natural tendencies may have a differential impact on subsequent measures.

Although the obtained results seem promising, they need further verification, as the methods used in the study are novel. Knowledge on how to activate particular TPs is lacking and, although the method proposed by our laboratory seems to be efficient, we feel that further studies on developing other ways of TP activation would be valuable. For example, it would be worthwhile to create more real-life methods of Time Perspective manipulations, such as especially created commercials or doctored fragments of news programs. Furthermore, it has to be noted that the financial decisions we analyzed were hypothetical and we relied on self-reported data. As a result, we obtained only quasi-behavioral data and have no information on actual financial decisions. Nevertheless, numerous studies show that the results of experiments with hypothetical rewards validly apply in everyday life (Johnson and Bickel, 2002; Kühberger et al., 2002; Locey et al., 2011). Moreover, the baseline level of people’s risk preference (risk preference as a trait) would be worth including in the further studies. It is also worth noticing that the short version (15 items) of the Zimbardo Time Perspective Inventory was used to measure Time Perspective as a trait. Meanwhile, the standard version of the questionnaire produces better reliability scores. However, it is much longer (56 items) and, due to technical reasons, we decided to use the shorter measure. Nevertheless, the robustness of the results indicate that the effects obtained it the study are reliable.

Concluding, the present study confirmed that Future and Present Hedonistic TPs influence consumers’ investing decisions. Nonetheless, it revealed that the nature of the relationship between chronic and situational levels of these TPs is not straightforward. The effect of activation of a particular TP will depend not only on the chronic level of this TP but also on chronic levels of other TPs.

## Author Contributions

KS, DM, and JR-W planned the research. KS and DM designed the study. KS analyzed the data. KS and JR-W wrote the manuscript along with inputs from DM during revisions.

## Conflict of Interest Statement

The authors declare that the research was conducted in the absence of any commercial or financial relationships that could be construed as a potential conflict of interest.

## Footnotes

1 Please contact the KS to obtain the English version of the manipulation task.

2 The sentences used to activate situational TPS were chosen based on the results of the pilot study. A total of 115 Polish adults took part in the pilot study. They were randomly assigned to one of the experimental conditions, where each TP was induced. Participants read two sets of three sentences each and were then asked to recall the sentences they had just read and write the first three associations that came into their minds. We expected that the associations would be related to the TP that was supposed to be activated. We marked all responses where all three associations were related to the TP or where two associations were related and the third was neutral as correct and counted them. One hundred and thirteen participants’ associations were consistent with our assumptions, therefore we decided to use the experimental material in the main study in an unchanged form.

3 We recruited twice as many participants to the control than to the experimental groups to be able to find enough people in the control group who were similar to those from the experimental groups in terms of their level of chronic Future and Present Hedonistic TPs. For each data analysis, we randomly chose participants from those who met the criteria of the TPs in the control group.

## Abbreviations

SZTPI: short version of the Zimbardo Time Perspective Inventory
TP: Time Perspective.
